# Salmagundi

**Published:** 1885-10

**Authors:** 


### SALMAGUNDI.

EDITED BY E. G. BETTY, D. D. S.

A third American edition of Foster's Physiology has lately been issued.

"DR. Herine Retsof, an eminent oculist, says that the common electric light produces color-blindness."

DR. FEHLING, of Stuttgart, known for his invention of the celebrated sugar test which bears his name, is dead.

TEN parts of collodion added to fifteen parts of creosote form a firm gelatinous mass of great efficacy, as a stopping and protective to exposed nerve-substance in dental cavities.—*Exchange.*

A few days ago we saw in the mouth, an upper right central incisor pivot tooth, that had been in use continuously for twenty-three years. It is mounted on the old-fashioned hickory pivot and in all the years mentioned, had been reset but once.

"To clean the teeth use a mixture of emery and sweet oil, following it with plenty of kerosene." This would seem to be queer advice, but as it is taken from a machinist's magazine, and from a chapter relating to circular saws, we have no doubt it is written in good faith.

THE healthfulness and immunity from diseases enjoyed by inhabitants of the neighborhood of pine forests is largely due to the continual presence of peroxide of hydrogen in the air. These districts have also been noticed to have a remarkably beneficial effect on the voice.—*Exchange.*

---

DR. H. A. SMITH, of Cincinnati, has lately ensconced himself and family in his new office and residence on West 8th street, where, no doubt, he will be pleased to show his friends around. When the cold weather comes he will start up the fires and have a warm house, we had almost said a house-warming.

---

AN ANTISEPTIC TOOTH-WASH.

℞ Sodæ boratis.............................. gr. xv

Thymol........................................ gr. vij

Aq. dest...................................... ℥ xvj

Dissolve.—*Magitot.*

---

DR. BURROUGHS, in the *Therapeutic Gazette*, advances the theory that, medically considered, nitro-glycerine is preferable to brandy, or alcohol in other forms as a stimulant. We do not know but that for a domestic blow-up or other kind of social explosions, nitro-glycerine might advantageously be employed as a substitute for brandy, whiskey, or even beer.—*Daily Paper.*

---

IT has often been remarked that during years of general business and financial depression, the classes at medical schools have been largely increased. The opinion seems to be that when business is dull, the place to go is the college where one can learn how to make money without working for it, and at times when nothing else pays.

---

"WE are still doing business at the old stand" and shall be glad to hear from all our friends, both old and young, long and short, fat and lean; for, as it takes all kinds of people to make a world, so does it take all sorts of interesting odds and ends, squibs and punches to make *Salmagundi* continue to be as engaging and coy as of yore. Ah there.

COVINGTON, KY., Sept. 12th, 1885.

EDITOR SALMAGUNDI:

The alleged "another new metal," whose name you wish to ascertain, alluded to by September Salmagundi as discovered by Dr. T. Dahl in a specimen of nickel ore from Krageroe, Norway, is known as *Norwegium.*

Truly yours,

J. S. CASSIDY.

---

THE first meeting of the *re-organized* Ohio State Dental Society, last Wednesday of this month, at Chillicothe, bids fair to be a grand one and the hope of everyone is, that the Society will from this time on resume its old-time usefulness and again be prominent in the dental councils of the land. Let every dentist of the State make an effort either to be present or send something to help along the cause of advanced dentistry, in that way he will honor himself.

---

A too liberal use of italics is an insult to the intelligence of the reader. A correspondent has a two-page article in the September number of the *Items of Interest* in which there are no less than forty-two italicised words. Whether this is the fault of the writer or the editor, we do not know, but, at least, it displays a lack of confidence in the reader, to say nothing of the fact that such means are frequently employed to give prominence to certain parts so that the reader will not notice the dearth of ideas.

---

SOUTHERN CONJURERS AND NORTHERN QUACKS.—Oliver Wendell Holmes has said that there are three classes of patients; One class will get well in spite of the doctor, another will die anyway, and the third, which is small in numbers, may be benefited by the doctors. Those patients of the conjure doctor who get well belong to the first class, those who die belong to the second, but none of his patients ever belong to the third class. After all the distinction of color so far as quacks are concerned, is not great. Some of our New England empirics are on a level with the Southern conjure doctors.—*Boston Budget.*

### A LESSON IN PHILOLOGY.

A YANK.

A maiden whose molar was sore
Walked into a tooth doctor's door;
    The tooth was yanked out
    And she flopped with a shout
From the soft-cushioned chair to the floor.

A YANK-EE.

The maiden shrieked loudly with dread,
And shook from her foot to her head;
    Her blood she thought blue;
    The truth now she knew,
For she saw it was awfully red.

A YANK-ER.

The dentist stood taking his ease,
Examined the tooth for disease;
    Then he offered his hand
    To assist her to stand,
And he said, "Fifty cents if you please."

—*Columbus Times.*

